# Virulent Poxviruses Inhibit DNA Sensing by Preventing STING Activation

**DOI:** 10.1128/JVI.02145-17

**Published:** 2018-04-27

**Authors:** Iliana Georgana, Rebecca P. Sumner, Greg J. Towers, Carlos Maluquer de Motes

**Affiliations:** a Department of Microbial Sciences, University of Surrey, Guildford, United Kingdom; b Division of Infection and Immunity, University College London, London, United Kingdom; University of Southern California

**Keywords:** DNA sensing, STING, innate immunity, poxvirus, vaccinia virus

## Abstract

Cytosolic recognition of DNA has emerged as a critical cellular mechanism of host immune activation upon pathogen invasion. The central cytosolic DNA sensor cGAS activates STING, which is phosphorylated, dimerizes and translocates from the endoplasmic reticulum (ER) to a perinuclear region to mediate IRF-3 activation. Poxviruses are double-stranded DNA viruses replicating in the cytosol and hence likely to trigger cytosolic DNA sensing. Here, we investigated the activation of innate immune signaling by 4 different strains of the prototypic poxvirus vaccinia virus (VACV) in a cell line proficient in DNA sensing. Infection with the attenuated VACV strain MVA activated IRF-3 via cGAS and STING, and accordingly STING dimerized and was phosphorylated during MVA infection. Conversely, VACV strains Copenhagen and Western Reserve inhibited STING dimerization and phosphorylation during infection and in response to transfected DNA and cyclic GMP-AMP, thus efficiently suppressing DNA sensing and IRF-3 activation. A VACV deletion mutant lacking protein C16, thought to be the only viral DNA sensing inhibitor acting upstream of STING, retained the ability to block STING activation. Similar inhibition of DNA-induced STING activation was also observed for cowpox and ectromelia viruses. Our data demonstrate that virulent poxviruses possess mechanisms for targeting DNA sensing at the level of the cGAS-STING axis and that these mechanisms do not operate in replication-defective strains such as MVA. These findings shed light on the role of cellular DNA sensing in poxvirus-host interactions and will open new avenues to determine its impact on VACV immunogenicity and virulence.

**IMPORTANCE** Poxviruses are double-stranded DNA viruses infecting a wide range of vertebrates and include the causative agent of smallpox (variola virus) and its vaccine vaccinia virus (VACV). Despite smallpox eradication VACV remains of interest as a therapeutic. Attenuated strains are popular vaccine candidates, whereas replication-competent strains are emerging as efficient oncolytics in virotherapy. The successful therapeutic use of VACV depends on a detailed understanding of its ability to modulate host innate immune responses. DNA sensing is a critical cellular mechanism for pathogen detection and activation of innate immunity that is centrally coordinated by the endoplasmic reticulum-resident protein STING. Here, STING is shown to mediate immune activation in response to MVA, but not in response to virulent VACV strains or other virulent poxviruses, which prevent STING activation and DNA sensing during infection and after DNA transfection. These results provide new insights into poxvirus immune evasion and have implications in the rational design of VACV-based therapeutics.

## INTRODUCTION

Cells sense the presence of invading pathogens by the use of pattern-recognition receptors (PRRs), a set of germ line encoded molecules recognizing pathogen-associated molecular patterns (PAMPs). Nucleic acids derived from viral infection are potent PAMPs that can be recognized in the cell cytosol and the endolysosomal compartment by dedicated PRRs. Recognition of RNA or DNA leads to the secretion of type I interferon (IFN) and other inflammatory cytokines and the expression of IFN-stimulated genes (ISGs), which restrict viral replication. Cytosolic double-stranded RNA is sensed by retinoic acid-inducible gene I (RIG-I) and melanoma differentiation-associated gene 5 (MDA-5). Both RIG-I and MDA-5 associate with the IFN-β promoter stimulator 1 (IPS-1; also known as MAVS), a protein residing in the mitochondrion that mediates the activation of IFN responsive factors (IRFs) and nuclear factor κ-light-chain-enhancer of activated B cells (NF-κB) (1). Cytosolic double-stranded DNA (dsDNA) can be sensed by multiple PRRs, the importance of which is largely dependent on the cell type (1). A critical sensor is the cyclic GMP-AMP synthase (cGAS), a nucleotidyltransferase that generates cyclic GMP-AMP (cGAMP) upon binding to dsDNA (2–4). cGAMP acts as a secondary messenger and docks on the stimulator of interferon genes (STING), inducing conformational changes and STING self-association. Upon cGAMP binding, STING is phosphorylated and activated and acts as a scaffold for the recruitment of TANK-binding kinase 1 (TBK-1) and the downstream activation of IRFs and NF-κB (5–8). Besides cGAS, other molecules have been proposed to recognize cytosolic DNA and contribute to STING-dependent IFN responses, including DAI (9); IFI16 (10); the DExD/H-box helicases DHX9, DDX36, (11), and DDX41 (12); and the DNA damage proteins Ku70/80 (13), DNA-PK (14), and Mre11 (15). Whether and how these molecules impact the cGAS-cGAMP-STING axis and whether they show pathogen and/or cell type specificity are important questions in the field (16, 17).

Poxviruses are a highly successful family of viruses infecting a broad range of species. Besides vaccinia virus (VACV), the prototypic member of the family and the virus that was used to eradicate smallpox, there are other poxviruses that can cause disease in humans (e.g., the monkeypox virus or the cowpox virus [CPXV]) or in animals (e.g., the rabbit myxoma virus or the mouse ectromelia virus [ECTV]). In addition to virulent strains, nonvirulent strains also exist and are popular candidates as recombinant vaccine vectors. Modified vaccinia virus Ankara (MVA) is an attenuated VACV strain generated through more than 500 serial passages in chicken cells. Through that serial passaging MVA lost its ability to replicate in human cells due to severe deletions and truncations affecting expression of multiple genes compared to the reference strains Copenhagen (COP) or Western Reserve (WR) (18, 19). Accordingly, and in contrast to COP or WR, which retain full inhibitory capacity, MVA has been shown to trigger innate immune activation in multiple experimental settings.

Poxviruses contain large dsDNA genomes of about 200 kbp with a coding capacity for more than 200 proteins. Poxviruses are unique in being the only dsDNA viruses to replicate exclusively in the cell cytoplasm. Replication in this compartment, however, makes this family of viruses particularly susceptible to detection by cytosolic DNA sensors. The success of poxviruses therefore implies the evolution of countermeasures to either avoid recognition or to dampen DNA sensing-induced innate immune activation. An example of such countermeasures is protein C16, which targets DNA-PK and inhibits IRF-3 activation in response to DNA (20). C16 contributes to virulence and is conserved in most VACV strains and poxviruses including variola virus, but is nonfunctional in MVA (20, 21). Besides C16, VACV encodes a vast array of immunomodulatory proteins able to inhibit the activation of the IRFs and NF-κB transcription factors (22). Some of these proteins exert their inhibitory action downstream of STING at the level of TBK-1 (23, 24) or IRF-3 (25) and can potentially block STING-mediated induction of type I IFN. However, no VACV proteins have been reported to specifically target the cGAS-cGAMP-STING axis. To seek evidence for such inhibitors, we investigated the differential capacities of four VACV strains to inhibit innate immunity and STING activation in a human monocytic cell line in response to DNA sensing. We demonstrate that virulent VACV strains, as well as CPXV and ECTV, but not the nonvirulent strain MVA, encode factors preventing STING phosphorylation and dimerization during viral infection and upon transfection with exogenous DNA. These findings uncover a novel immune evasion strategy in poxviruses and highlight the importance of DNA sensing in the innate antiviral defense.

## RESULTS

### PMA-differentiated THP-1 cells activate IRF-3 in response to MVA, but not COP or WR, infection.

**FIG 1:**
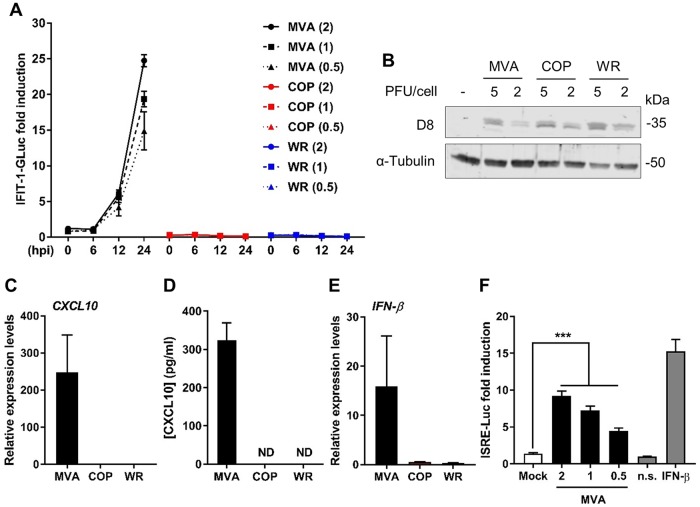
MVA, but not COP or WR, activate IRF-3 and IFN production in PMA-differentiated THP-1 cells. (A) PMA-differentiated THP-1-IFIT-1-GLuc cells were infected in quadruplicate with the indicated PFU/cell (in parentheses) of MVA (black), COP (red), or WR (blue), and the medium was analyzed for luciferase activity at the indicated times postinfection. Data were normalized to mock-infected samples and are presented as the fold increase. (B) BHK-21 cells were infected with the indicated PFU/cell of MVA, COP, and WR. Cells were lysed in RIPA buffer, and whole-cell lysates were subjected to SDS-PAGE and immunoblotting for D8 and α-tubulin. (C and E) PMA-differentiated THP-1-IFIT-1-GLuc cells were infected in triplicate with 2 PFU/cell of MVA, COP, or WR, and 16 h later the mRNA expression levels of *CXCL10* (C) and *IFN-β* (E) were assessed by qPCR. (D) Medium from cells infected as described above with MVA, COP, and WR were subjected to ELISA against CXCL10. (F) Medium from cells infected with 2, 1, and 0.5 PFU/cell of MVA or uninfected was transferred to HEK293T previously transfected with an ISRE reporter plasmid (ISRE-FLuc) and a renilla luciferase plasmid (RLuc). Plain medium (n.s.) and medium containing recombinant IFN-β (25 ng/ml) were also used as controls. FLuc/RLuc ratios were normalized to the n.s. control and are presented as a fold increase. In all assays, data are presented as means ± the standard deviations (SD) and show the results from one representative experiment of at least three. *, *P* < 0.05; **, *P* < 0.01; ***, *P* < 0.001 (unpaired Student *t* test).

To assess the capacity of VACV strains to modulate cellular innate immunity, we sought a cell line that (i) responds to PAMPs, (ii) provides consistent and high-throughput quantitative measurements, and (iii) is amenable to genetic manipulation. THP-1 monocytes expressing Gaussia luciferase (GLuc) under the control of the promoter of the IRF-3-dependent gene *IFIT-1* (26) provided these characteristics. We differentiated these cells with phorbol 12-myristate 13-acetate (PMA) for 48 h and infected them with three different VACV strains at several PFU per cell. We monitored GLuc activity over a period of 24 h and plotted it as a fold increase over mock-infected cells. MVA infection triggered IFIT-1-driven GLuc activity, and this was quantitated 24 h postinfection (p.i.) at approximately 25-, 20-, and 15-fold increases after infection with 2, 1, and 0.5 PFU/cell, respectively (Fig. 1A). Infection with a higher PFU/cell did not yield higher levels of activation (data not shown), possibly due to MVA-induced apoptosis (27, 28). Conversely, infection with VACV strains COP and WR did not induce GLuc activity. To confirm that these differences were not caused by variations in virus titer, the same sucrose purified stocks were used to infect permissive BHK-21 cells with 5 and 2 PFU/cell. At 12 h p.i., the cells were lysed, and the lysates were subjected to SDS-PAGE. Immunoblotting against the late viral protein D8 confirmed that infection levels were similar across the different strains (Fig. 1B). We then repeated the infection of THP-1 cells with 2 PFU/cell and measured the induction of *CXCL10* and *IFN-β* mRNA by quantitative PCR at 24 h p.i. MVA infection triggered *CXCL10* expression, whereas COP and WR infections did not (Fig. 1C). The production of CXCL10 was subsequently confirmed by enzyme-linked immunosorbent assay (ELISA) (Fig. 1D). Similarly, we detected a significant increase in *IFN-β* mRNA expression in response to MVA, but not COP or WR, infection (Fig. 1E). The presence of active IFN-β in the MVA-infected medium was confirmed in a bioassay on HEK293T cells transfected with a reporter expressing luciferase under the control of the IFN-stimulated response element (ISRE). Supernatants from MVA-infected THP-1 cells induced a statistically significant increase in ISRE activity in the HEK293T cells in a dose-dependent manner (Fig. 1F). From these data we concluded that MVA induces a robust innate immune response in THP-1-IFIT-1-GLuc cells that is not observed with COP or WR and that measurements of GLuc activity correlate with the upregulation of antiviral cytokines, including type I IFN.

### IRF-3 activation in response to MVA infection requires the cGAS-STING axis.

**FIG 2:**
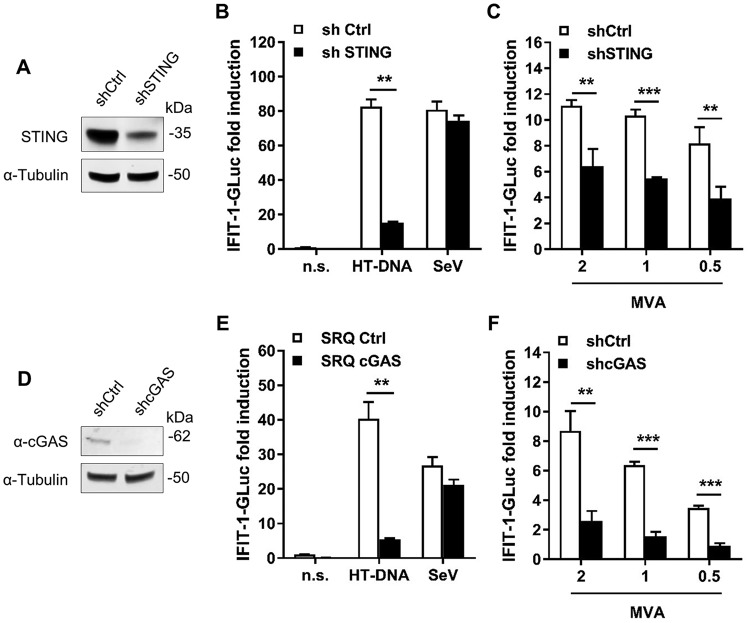
Activation of IRF-3 in THP-1 cells upon MVA infection requires STING and cGAS. (A and D) Whole-cell lysates from THP-1 cells transduced with lentivirus expressing shRNA against STING (shSTING) or control (shCtrl) (A) or against cGAS (shcGAS) or control (shCtrl) (D) were subjected to immunoblotting against α-tubulin and STING (A) or cGAS (D). (B and E) PMA-differentiated shSTING or shCtrl (B) or shcGAS or shCtrl (E) THP-1 cells were transfected with HT-DNA or infected with Sendai virus (SeV). After 16 h, the GLuc activity in the supernatant was measured and is presented as a fold increase over nonstimulated conditions (n.s.). (C and F) PMA-differentiated shSTING or shCtrl (C) or shcGAS or shCtrl (F) THP-1 cells were infected with 2, 1, and 0.5 PFU/cell of MVA. After 24 h, the GLuc activity was measured and is presented as a fold increase over a mock-infected condition. Data in all graphs are presented as means ± the SD, and the results of one representative experiment of at least three, each performed in triplicate, are shown. **, *P* < 0.01; ***, *P* < 0.001 (unpaired Student *t* test for the indicated comparisons).

VACV is a dsDNA virus that replicates in the cell cytoplasm and therefore has the potential to be detected by cytosolic DNA sensing mechanisms, in which STING has a pivotal role. To determine whether STING had an impact on the IRF-3 response induced by VACV in THP-1 cells, we transduced THP-1-IFIT-1-GLuc cells with a lentivirus expressing shRNA against cellular STING, or a control shRNA, and selected them with puromycin. Control and shSTING cells were PMA-differentiated and STING levels assessed by immunoblotting. shSTING cells showed a reduction in STING expression compared to control cells (Fig. 2A). Furthermore, cells were exposed to cytosolic DNA (herring testes [HT]-DNA transfection) or RNA (Sendai virus [SeV] infection) and IRF-3 responses were determined by luciferase activity. shSTING cells had a significantly impaired response to HT-DNA transfection compared to control cells, but both cell lines responded equally to SeV infection which activates IRF-3 via RNA sensors (Fig. 2B). We next measured the response to VACV infection in the STING-depleted cells. Differentiated cells were challenged with MVA at various PFU/cell, and the luciferase activity was measured at 24 h p.i. MVA infection triggered IFIT-1-driven GLuc expression in a dose-dependent manner, and this was impaired in shSTING cells in a statistically significant manner (Fig. 2C).

To further confirm that MVA-induced activation of IRF-3 in THP-1 cells derived from DNA sensing, we generated a THP-1 cell line expressing shRNA against cGAS, the main cellular DNA sensor. Immunoblotting for cGAS confirmed cGAS depletion in shcGAS cells compared to control cells (Fig. 2D). Accordingly, shcGAS cells failed to induce IFIT-1-GLuc expression in response to HT-DNA transfection while their response to the RNA sensing activator SeV was unaffected (Fig. 2E). Similar to the results obtained in cells depleted for STING, MVA infection triggered a significantly reduced response in cells depleted for cGAS (Fig. 2F). Together, these results demonstrate that cGAS and STING contribute to IRF-3 activation in THP-1 cells infected with MVA.

### COP and WR suppress IRF-3 activation induced by exogenous DNA.

**FIG 3:**
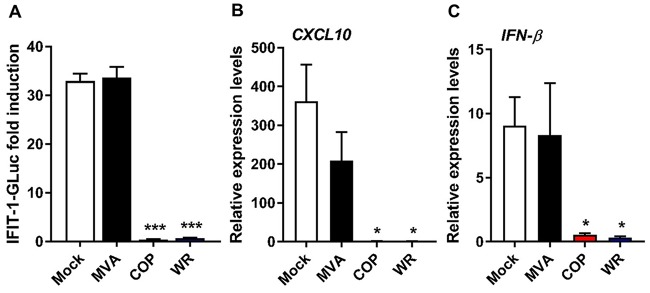
VACV strains COP and WR inhibit IRF-3 activation in response to exogenous DNA. PMA-differentiated THP-1 cells were infected with 2 PFU/cell of MVA, COP, or WR for 6 h and subsequently transfected with HT-DNA for a further 16 h. (A) The GLuc activity was measured and is presented as a fold increase over a mock-infected condition. (B and C) Cells were infected as in panel A and subsequently transfected with HT-DNA for a further 6 h. The *CXCL10* and *IFN-β* mRNA expression levels were assessed by qPCR. Data are presented as means ± the SD, and the results of one representative experiment of at least three, each performed in triplicate, are shown. *, *P* < 0.05; ***, *P* < 0.001 (unpaired Student *t* test comparing infections with mock infections).

The absence of IRF-3 activation upon COP or WR infection could be explained by (i) an ability of these viruses to mask their dsDNA genome in a manner that is lost in MVA, or (ii) the production of viral factors that prevent IRF-3 activation. We addressed the presence of viral factors by determining the capacity of all three VACV strains to inhibit IRF-3 activation mediated by exogenous DNA. First, PMA-differentiated THP-1 cells were infected with 2 PFU/cell of MVA, COP, and WR for 6 h and subsequently transfected with HT-DNA. After 16 h, HT-DNA transfection induced an ∼30-fold increase in IFIT-1-driven GLuc activity in mock-infected and MVA-infected cells, and this was completely suppressed in COP-infected and WR-infected cells (Fig. 3A). We then repeated the infection and measured *CXCL10* and *IFN-β* mRNA expression 6 h after HT-DNA transfection. DNA challenge triggered expression of *CXCL10* (Fig. 3B) and *IFN-β* (Fig. 3C) in mock-infected cells, and this was similar to that observed in MVA-infected cells. COP and WR infections inhibited the DNA-induced expression of both cytokines in a statistically significant manner. Therefore, COP and WR efficiently abolish IRF-3 activation in response to exogenous DNA in THP-1 cells, most likely by the expression of viral factors that are lost or defective in MVA.

### COP and WR inhibit STING phosphorylation in response to DNA.

**FIG 4:**
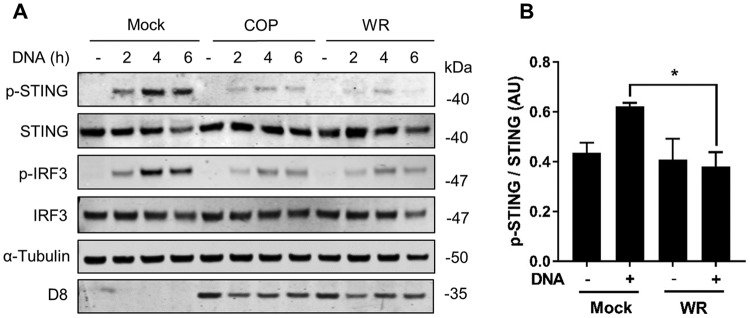
VACV strains COP and WR inhibit STING phosphorylation in response to DNA transfection. (A) PMA-differentiated THP-1 cells were infected with 2 PFU/cell of the indicated viruses for 6 h and subsequently transfected with HT-DNA (4 μg/ml) for the indicated time. Cells were lysed in RIPA buffer, and whole-cell lysates were subjected to SDS-PAGE and immunoblotting against the indicated proteins. (B) Ratio of phosphorylated/total STING for the indicated conditions integrating quantitative data from three independent experiments. AU, arbitrary units. *, *P* < 0.05 (unpaired Student *t* test for the indicated comparison).

The ability of VACV virulent strains to block DNA-induced IRF-3 signaling could be ascribed not only to a cumulative effect of viral inhibitors acting on the IRF-3 pathway but also to the presence of specific inhibitors acting at the level of cGAS/STING. To address the latter, we examined DNA-induced STING activation in VACV-infected cells. A hallmark of STING activation is the phosphorylation of Ser^366^, an event associated with IRF-3 recruitment and activation (6). We infected PMA-differentiated THP-1 cells with COP or WR for 6 h and subsequently transfected the cells with HT-DNA. Whole-cell lysates were subjected to immunoblotting against p-STING Ser^366^ at 2, 4, and 6 h after DNA transfection. In mock-infected cells, p-STING was detected at 2 h and was prominent at 4 and 6 h posttransfection, concomitant with p-IRF-3 induction (Fig. 4A). In infected cells, however, p-STING levels were significantly reduced, particularly after WR infection, and this correlated with a reduction in p-IRF-3. Immunoblotting against viral protein D8 revealed that the infection levels between COP and WR were similar. Phosphorylated and total STING levels from three independent experiments using WR were quantitated, and the reduction of p-STING upon DNA challenge was shown to be statistically significant (Fig. 4B).

### COP and WR inhibit STING dimerization.

**FIG 5:**
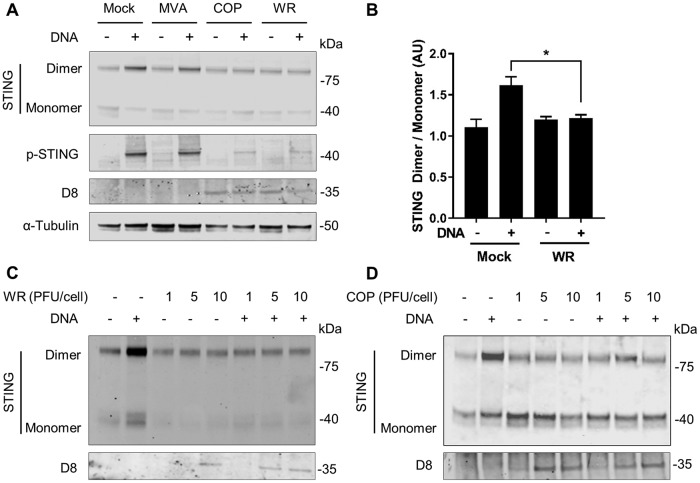
VACV strains COP and WR inhibit STING dimerization in response to DNA transfection. (A) PMA-differentiated THP-1 cells were infected with 2 PFU/cell of the indicated viruses for 6 h and subsequently transfected with HT-DNA for a further 4 h. Cells were lysed in RIPA buffer, and whole-cell lysates were subjected to SDS-PAGE and immunoblotting against the indicated proteins. (B) Ratio of dimeric/monomeric STING for the indicated conditions integrating quantitative data from 3 independent experiments. AU, arbitrary units. *, *P* < 0.05 (unpaired Student *t* test for the indicated comparison). (C and D) PMA-differentiated THP-1 cells were infected with the indicated PFU/cell of WR (C) or COP (D) for 6 h and subsequently transfected with HT-DNA. Cell lysates were subjected to SDS-PAGE and immunoblotting as in panel A.

To further assess whether WR and COP suppress STING activation, we examined the ability of these viruses to block STING dimerization in response to DNA (6, 7, 29). Cells were infected with MVA, COP, or WR at 2 PFU/cell and subsequently transfected with HT-DNA for a further 4 h. Cells were lysed and treated to preserve endogenous STING dimers, and these were assessed by immunoblotting. In nonstimulated, mock-infected cells a band at ∼80 kDa corresponding to the expected size of a STING dimer was detected and became more intense upon DNA sensing stimulation, an observation consistent with the formation of STING dimers in response to exogenous DNA (Fig. 5A). DNA-induced STING dimerization in MVA-infected cells was indistinguishable from that in mock-infected cells, highlighting the inability of MVA to block STING activation. This notion was also supported by measuring p-STING levels. Conversely, cells infected with COP or WR showed a reduction in DNA-induced STING dimerization. In agreement with previous observations, p-STING levels were also efficiently suppressed by COP and WR. The levels of viral D8 confirmed similar infectivity between COP and WR. D8 could not be detected in MVA-infected cell lysates due to the fact that MVA does not express late proteins in THP-1 cells. We then repeated this experiment and quantitated the dimeric and monomeric STING bands upon WR infection and DNA stimulation. The results confirmed that WR infection inhibited STING dimerization in a statistically significant manner (Fig. 5B). Finally, we assessed STING dimerization and stability upon WR infection at multiple PFU/cell. Infection with as little as 1 PFU/cell was sufficient to prevent DNA-induced STING dimerization (Fig. 5C). Infection with a higher PFU/cell did not affect the levels of monomeric STING, which remained largely constant and close to those seen in unstimulated cells. Similar data were obtained with COP (Fig. 5D). This suggests that VACV does not affect STING stability. Taken together, these data demonstrate for the first time that VACV prevents STING phosphorylation and dimerization in response to DNA.

### The VACV deletion mutant vv811 inhibits STING activation.

**FIG 6:**
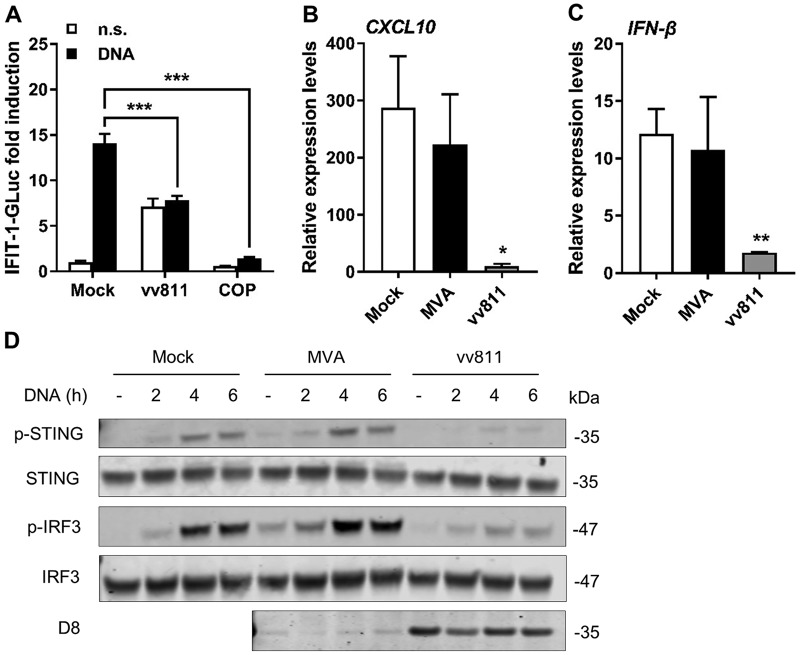
vv811 inhibits STING activation. (A) PMA-differentiated THP-1 cells were infected with 2 PFU/cell of MVA, vv811, or COP for 6 h and subsequently transfected with HT-DNA for a further 16 h. The GLuc activity was measured and is presented as a fold increase over mock-infected conditions. (B and C) Cells were infected as in panel A and subsequently transfected with HT-DNA for a further 6 h. The *CXCL10* and *IFN-β* mRNA expression levels were assessed by qPCR. (D) PMA-differentiated THP-1 cells were infected with 2 PFU/cell of the indicated viruses for 6 h and subsequently transfected with HT-DNA (4 μg/ml) for a further 4 h. Cells were lysed in RIPA buffer, and whole-cell lysates were subjected to SDS-PAGE and immunoblotting against the indicated proteins. The results of one representative experiment of at least three performed are shown. Data in all graphs are presented as means ± the SD, each performed in triplicate. *, *P* < 0.05; **, *P* < 0.01; ***, *P* < 0.001 (unpaired Student *t* test comparing infection to mock infection).

VACV protein C16 is expressed by COP and WR and targets DNA-PK (20), a cytosolic DNA sensor recognizing VACV and acting via STING (14, 30). To determine whether C16 was responsible for the observed STING inhibition, we took advantage of vv811, a VACV deletion mutant deriving from COP that lacks 55 open reading frames (ORFs), including *C16L* (31, 32). In contrast to COP, infection with vv811 triggered ∼7-fold increase in IFIT-1-driven GLuc activity (Fig. 6A), suggesting that the absence of immunomodulatory genes limits the ability of vv811 to block IRF-3 responses in differentiated THP-1 cells. vv811 infection reduced the levels of IRF-3 activation observed after DNA transfection compared to mock-infected cells, and this was statistically significant (Fig. 6A). However, vv811 did not reduce IRF-3 activation as effectively as COP, but rather to the levels induced by vv811 infection in the absence of DNA stimulation, suggesting that this baseline activation on infection may be induced by a response that is unrelated to DNA sensing. vv811 inhibition of DNA-induced IRF-3 activation was also observed on measurement of *CXCL10* (Fig. 6B) and *IFN-β* (Fig. 6C) mRNA expression. We then assessed the kinetics of activation of p-STING after HT-DNA transfection in cells infected with vv811 (Fig. 6D). As shown previously, mock-infected cells showed substantial levels of p-STING at 4 and 6 h poststimulation, and these correlated with p-IRF-3 levels. The kinetics of DNA-induced p-STING formation in MVA-infected cells was indistinguishable from that observed in mock-infected cells, implying that no inhibitors of DNA sensing are expressed in MVA. Indeed, low levels of p-STING could be detected after MVA infection in the absence of exogenous DNA transfection, indicating that MVA infection is sufficient to trigger STING activation. In contrast, vv811 infection efficiently suppressed both p-STING and p-IRF-3. As expected, viral D8 could not be detected in MVA-infected cells due to its late expression, but it was detected in vv811-infected cells, demonstrating that this virus replicates in THP-1 cells despite its multiple deletions. Thus, it appears that VACV expresses mechanisms, other than C16, to prevent STING activation in response to DNA sensing.

### VACV inhibits STING activation in response to cGAMP.

**FIG 7:**
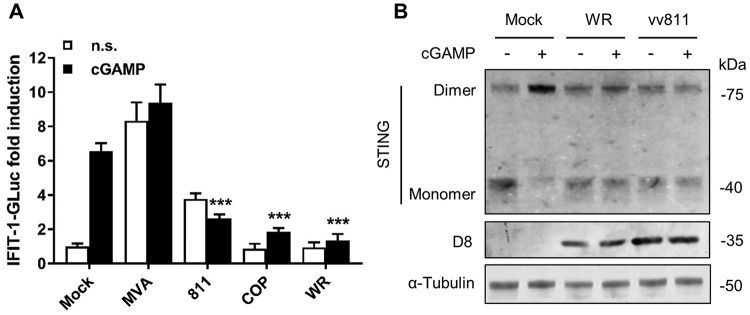
VACV inhibits STING activation in response to cGAMP. (A) PMA-differentiated THP-1 cells were infected with 2 PFU/cell of the indicated viruses for 6 h and subsequently exposed to cGAMP a further 16 h. The GLuc activity was measured and is presented as a fold increase over mock-infected conditions. Data are presented as means ± the SD, each performed in triplicate. ***, *P* < 0.001 (unpaired Student *t* test comparing cGAMP-stimulated infections to cGAMP-stimulated mock infections). (B) PMA-differentiated THP-1 cells were infected with 2 PFU/cell of the indicated viruses for 6 h and subsequently exposed to cGAMP (15 μg/ml) for a further 6 h. Cells were lysed in RIPA buffer, and whole-cell lysates were subjected to SDS-PAGE and immunoblotting against the indicated proteins. The results of one representative experiment of at least two are shown.

To gain further insight into VACV inhibition of STING-dependent DNA sensing signaling, we assessed the inhibitory capacity of the different VACV strains in response to cGAMP. Exposure to cGAMP triggered an ∼6-fold increase in IRF-3-driven reporter activity in PMA-differentiated THP-1 cells (Fig. 7A). This activation was significantly suppressed in cells that had been previously been infected with vv811, COP, or WR. Infection with MVA triggered reporter activity on its own, and this activity was even enhanced after exposure to cGAMP. To further demonstrate that cGAMP activated STING and that this was suppressed by VACV inhibitory strains, we assessed STING dimerization in response to cGAMP. Exposure to cGAMP induced the formation of STING dimers in mock-infected cells, but these were inhibited in cells infected with WR and even with the deletion mutant vv811 (Fig. 7B). Therefore, irrespective of the potential ability to interfere with cGAS and/or its enzymatic activity, VACV efficiently antagonizes the action of STING.

### Cowpox and Ectromelia virus prevent STING activation.

To determine whether inhibition of STING activation is unique to VACV or occurs after infection with other poxviruses, we studied CPXV and ECTV. Cells were infected with 2 PFU/cell of CPXV reference strain Brighton Red and ECTV reference strain Moscow, as well as WR as a control, for 6 h and subsequently stimulated by DNA transfection for a further 4 h. The levels of STING dimerization were assessed by immunoblotting, and viral infection was confirmed by detecting D8 (Fig. 7). As expected, uninfected cells stimulated with DNA showed elevated levels of STING dimers, and these were absent in WR-infected cells. Infection with CPXV and ECTV strains also inhibited STING dimerization to the same extent as WR, indicating that inhibition of STING activation is conserved among multiple orthopoxviruses.

## DISCUSSION

**FIG 8:**
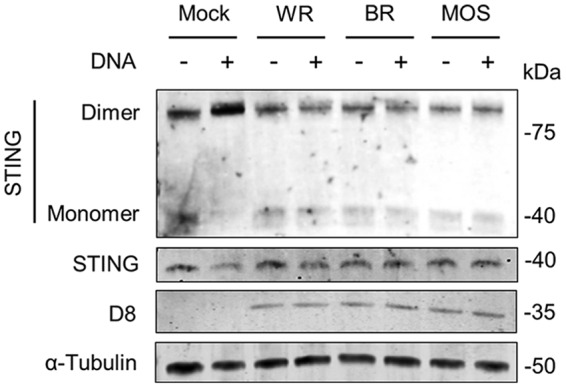
CPXV strain Brighton Red (BR) and ECTV strain Moscow (MOS) inhibit DNA-induced STING dimerization. PMA-differentiated THP-1 cells were infected with 2 PFU/cell of the indicated viruses for 6 h and subsequently transfected with HT-DNA for a further 4 h. Cells were lysed in RIPA buffer, and whole-cell lysates were subjected to SDS-PAGE and immunoblotting against the indicated proteins. The results of one representative experiment of at least three are shown.

STING has emerged as a pivotal molecule in the integration of signals deriving from various cytosolic DNA sensors and in particular cGAS. Upon binding cGAMP STING dimerizes, translocates to perinuclear structures, and is phosphorylated (5–8). This promotes the phosphorylation and activation of IRF-3 and the subsequent production of type I IFN and inflammatory cytokines. Whether and how VACV has evolved mechanisms counteracting the cGAS-STING axis is currently unknown. We provide evidence here demonstrating that VACV inhibits the phosphorylation and dimerization of STING in response to DNA and cGAMP (Fig. 4, 5, and 7). These observations correlated with the inhibition of IRF-3 activation and of the expression of IFN-β and CXCL10 cytokines (Fig. 1 and 3). Inhibition of STING activation occurred after infection with strains COP and WR, but not MVA. MVA is an attenuated VACV strain unable to replicate and express late genes in most mammalian cell types, including human cells. For this reason it is typically titrated on chicken cells (i.e., chicken embryo fibroblasts [CEF]) as opposed to virulent VACV strains which can be titrated on conventional mammalian cell lines (e.g., BS-C-1). Because of this discrepancy we infected permissive BHK-21 cells (in which MVA is fully replicative and hence the late viral protein D8 is expressed) in parallel to differentiated THP-1 cells and observed similar levels of infectivity across the three VACV strains (Fig. 1). In PMA-differentiated THP-1 cells, MVA infection was sufficient to trigger IRF-3 activation (Fig. 1), and this activation required cGAS and STING (Fig. 2). These data are in line with the reported role of cGAS upon MVA infection in murine dendritic cells (33). MVA is a VACV strain sharing the same dsDNA PAMP as COP or WR but containing up to six major genomic deletions (18). A likely explanation for the inability of MVA to block STING activation is that MVA does not express the viral gene(s) responsible for STING inhibition, which are present in COP and WR. However, the possibility existed that the COP and WR genomes were immunologically weaker PAMPs than that of MVA, or that COP and WR were able to mask their genomes more efficiently. Although these possibilities cannot formally be ruled out, the observation that COP and WR blocked STING activation in response to transfected DNA or cGAMP exposure (Fig. 4 and 7) argues that virulent VACV strains express one or multiple factors that are capable of acting in *trans* to block STING activation induced by the viral genome, as well as exogenous DNA. These factors may not operate in MVA, but they (or functionally equivalent products) must be present in CPXV and ECTV, and presumably in other virulent poxviruses, because both ECTV and CPXV inhibited STING activation to the same extent as VACV (Fig. 8).

There are 29 genes absent in MVA due to large genomic deletions and truncations. In addition, MVA contains nonfunctional copies of genes due to point mutations and small truncations acquired during serial passage in chicken cells and does not express late genes due its inability to complete the viral cycle in most mammalian cells (19, 34, 35). Therefore, a large number of VACV proteins could account for the inhibition of STING activation reported here. An obvious candidate is protein C16, the viral inhibitor of DNA-PK, which is absent in MVA, but conserved across VACV replicative strains, as well as in CPXV and ECTV (21). Hence, we assessed DNA-induced STING activation during vv811 infection, a VACV deletion mutant that contains two large deletions in the genome terminal regions and does not encode C16 (31). vv811 infection was sufficient to trigger intermediate levels of IRF-3 activation, which is consistent with the loss of multiple immune modulators encoded by the missing genome fragments. Despite this reduced inhibitory capacity, vv811 was as effective as fully virulent VACV strains in preventing DNA-induced STING activation (Fig. 6). This indicates that neither C16 nor any of the other 54 ORFs missing in vv811 is required for the observed STING inhibition and that additional mechanisms must exist and cooperate with C16 to effectively block DNA sensing in multiple contexts. Identification of the viral mechanism(s) may thus require a combination of genetic and biochemical approaches and will be the subject of future studies.

Whatever the exact mechanism is, it will add to the inhibitory capacity provided by other VACV factors targeting the IRF-3 signaling cascade downstream of cGAS/STING such as proteins C6 and K7, which act at the level of TBK-1 (23, 24), or protein N2, which blocks IRF-3 activity in the nucleus (25). VACV redundancy in targeting cellular functions is common and also occurs to prevent NF-κB activation (22) or cell death (36). In the case of NF-κB, vv811 has been shown to inhibit NF-κB activation to the same extent as its parental strain COP despite its large genome deletions (37), and even a recombinant vv811 engineered to lack all described NF-κB inhibitors retained an inhibitory capacity similar to that of the original vv811, revealing the existence of additional, yet uncharacterized inhibitors (38–40). This remarkable redundancy is necessary to counteract the complex signaling and cross talk governing innate immune responses *in vivo* and indicates that in cells the contribution of each individual viral protein to the inhibition of a signaling cascade during infection can only be assessed by studying very specific events (i.e., STING phosphorylation and STING dimerization in this case).

Multiple human viruses target STING function. For instance, the human cytomegalovirus protein UL82 binds to STING and prevents its translocation and activation (41), the Kaposi's sarcoma-associated herpesvirus (KSHV) protein vIRF1 prevents STING association with TBK-1 (42), and the human papillomavirus and adenovirus suppress IFN-β production by targeting STING with their proteins E7 and E1A, respectively (43), whereas dengue virus does so by cleaving STING via protein NS2B/3 (44, 45). Cleavage is unlikely to underlie the VACV mechanism since our data did not indicate a reduction in STING stability during infection. Other human viruses target cGAS, e.g., the KSHV proteins ORF52 and LANA (46, 47). At present our data demonstrate that VACV targets STING but do not exclude that VACV may have evolved complementary mechanisms to interfere with cGAS/STING signaling, such as masking the viral DNA with DNA-binding proteins, targeting cGAS, or enzymatically degrading cGAMP. The wide array of mechanisms that viruses employ to inhibit the cGAS-STING axis highlights the crucial role of this pathway in innate antiviral defense. In addition, the cGAS-STING pathway has a critical role in inflammatory diseases and in the induction of effective anticancer adaptive immunity (48–50). STING activation is recurrently suppressed in a number of cancers, and the level of STING signaling has been shown to correlate with the outcome of VACV or herpesvirus-based oncotherapy (51). The activation and regulation of DNA sensing during poxvirus infection provides a unique model that offers a better mechanistic understanding of DNA-mediated activation of immune responses and that will contribute to the rational design of VACV-based therapies for vaccination and oncolytic treatment.

## MATERIALS AND METHODS

### Cells, reagents, and viruses.

HEK-293T, BS-C-1, RK-13, and BHK-21 cells were grown in Dulbecco modified Eagle medium (Life Technologies) supplemented with 10% heat-inactivated fetal calf serum (FCS; Seralab) and 100 U/ml penicillin plus 100 μg/ml streptomycin (Pen/Strep; Life Technologies). THP-1-IFIT-1-GLuc cells were a gift from Veit Hornung (University of Munich, Munich, Germany). These cells had been modified to express GLuc under the control of the *IFIT-1* promoter (26). THP-1-IFIT-1-GLuc cells were grown in RPMI 1640 (Life Technologies) supplemented with 15% FCS and Pen/Strep. PMA (Santa Cruz Biotechnology) was dissolved in dimethyl sulfoxide at 10 mg/ml. HT-DNA (Sigma) was dissolved in water at 2 mg/ml. 2′3′-cGAMP (Invivogen) was dissolved in water at 50 mg/ml. Sendai virus was a gift from Steve Goodbourn (St. George's University of London, London, United Kingdom). VACV strains MVA, vv811, COP, and WR, as well as CPXV strain Brighton Red, were obtained from Geoffrey L. Smith (University of Cambridge, Cambridge, United Kingdom). ECTV strain Moscow was from Antonio Alcami (Centro de Biología Molecular Severo Ochoa, Madrid, Spain). MVA was grown and titrated in CEF by conventional plaque assay. All other viruses were expanded in RK-13 or BS-C-1 cells and titrated in BS-C-1 cells. All viruses were purified through a 36% sucrose cushion before use.

### Reporter gene assays.

THP-1-IFIT-1-GLuc cells were seeded in 96-well plates at a density of 5 × 10^4^ cells per well in the presence of PMA (50 ng/ml). After 48 h, the cells were infected in quadruplicate with the indicated viruses in RPMI 1640 supplemented with 2% FCS at the indicated PFU per cell, and the medium was collected at the indicated times postinfection. When DNA stimulation was performed, PMA-differentiated cells were infected with the indicated viruses at 2 PFU/cell for 6 h and then transfected with HT-DNA at 0.5 μg/ml for a further 16 h. Transfections were performed in Opti-MEM (Life Technologies) in the presence of the transfection reagent *Trans*IT-LT-1 (Mirus Bio) according to the manufacturer's recommendations. When cGAMP stimulation was performed, cells were infected as described above and then exposed to cGAMP at 10 μg/ml in the medium for a further 16 h. The luciferase activity was measured in a Clariostar plate reader (BMG Biotech) in the presence of coelenterazine (NanoLigh Technology) at 2 μg/ml. Data were normalized to mock-infected samples and are presented as a fold increase.

### Quantitative PCR.

THP-1-IFIT-1-GLuc cells were seeded in 24-well plates at a density of 2 × 10^5^ cells per well in the presence of PMA (50 ng/ml). After 48 h, the cells were infected in triplicate with the indicated viruses in RPMI 1640 supplemented with 2% FCS at the indicated PFU/cell for 16 h, or for 6 h and subsequently transfected with HT-DNA for a further 6 h as described above. RNA was extracted using a Total RNA purification kit (Norgen Biotech) and transcribed into cDNA using Superscript III reverse transcriptase (Invitrogen) according to the manufacturer's protocol. cDNA was diluted 1:5 in water and used as a template for real-time PCR using SYBR green PCR master mix (Applied Biosystems) in a LightCycler 96 (Roche). Expression of each gene was normalized to an internal control (*18S*), and these values were then normalized to the nonstimulated mock-infected control cells to yield a fold induction. The primers used for *CXCL10* detection were as previously described (52). The primers used for *18S* detection (forward, 5′-GTAACCCGTTGAACCCCA-3′; reverse, 5′-CCATCCAATCGGTAGTAGG-3) and *hIFN-β* detection (forward, 5′-ACATCCCTGAGGAGATTAAGCA-3′; reverse, 5′-GCCAGGAGGTTCTCAACAATAG-3′) were as indicated here.

### ELISA.

Cell culture supernatants from virus-infected THP-1 cells grown in 24-well plates were assayed for CXCL10 using Duoset ELISA reagents (R&D Biosystems) according to the manufacturer's instructions.

### IFN bioassay.

THP-1-IFIT1-GLuc cells were seeded in 96-well plates at a density of 5 × 10^4^ cells per well in the presence of PMA (50 ng/ml). After 48 h, the cells were infected in quadruplicate with the indicated viruses in RPMI 1640 supplemented with 2% FCS at the indicated PFU/cell. The medium was collected 24 h later and transferred to 96-well plates containing HEK293T cells previously transfected for 24 h with 70 ng/well of a reporter plasmid expressing firefly luciferase under the control of ISRE (Promega) and 10 ng/well of a control plasmid expressing Renilla luciferase (RLuc; Promega) using polyethylenimine (PEI; Sigma) at a ratio of 1:2 (μg of DNA:μl of PEI). Activation with recombinant hIFN-β at 25 ng/ml was also included as a control. At 10 h after medium transfer or hIFN-β activation, the cells were lysed in passive lysis buffer (Promega). The FLuc and RLuc activities were measured in a Clariostar plate reader (BMG Biotech), and FLuc/RLuc ratios were calculated for each well. Data were normalized to mock-infected THP-1 samples and are presented as fold increases.

### Generation of cell lines depleted for cGAS and STING.

THP-1-IFIT-1-GLuc cells were first depleted for SAMHD1 using specific short hairpin sequences expressed from the HIV-1-based shRNA expression vector HIVSiren (53) and selected for hygromycin resistance (Invivogen, 200 μg/ml), followed by depletion for STING using the above HIVSiren system, or cGAS using the MLV-based shRNA expression vector pSIREN-RetroQ (Clontech), and selected for puromycin resistance (1 μg/ml; Merck Chemicals, Ltd.). Lentiviral particles were produced by transfection of HEK293T cells with either (i) 1.5 μg of pHIVSIREN shRNA, 1 μg of p8.91 packaging plasmid (54), and 1 μg of vesicular stomatitis virus-G glycoprotein expressing plasmid pMDG (Genscript) or (ii) 1.5 μg of pSIREN-RetroQ shRNA, 1 μg of pCMVi (MoMLV Gag-Pol), and 1 μg of pMDG using Fugene 6 transfection reagent (Promega) according to the manufacturer's instructions. Virus supernatants were harvested at 48 and 72 h posttransfection, pooled, and used to transduce THP-1-IFIT-1-GLuc cells. The sequences were as follows: SAMHD1 shRNA, 5′-CGGGCCATCATCTTGGAATCCAAACTCGAGTTTGGATTCCAAGATGATGGCTTTTT-3′; STING shRNA, 5′-GCCTGATAACCTGAGTATGTTCAAGAGACATACTCAGGTTATCAGGCTTTTTTACGCGT-3′; and cGAS shRNA, 5′-GGAAGGAAATGGTTTCCAATTCAAGAGATTGGAAACCATTTCCTTCCTTTTTTACGCGT-3′.

### SDS-PAGE and immunoblotting.

Cells were lysed in radioimmunoprecipitation assay (RIPA) buffer supplemented with protease and phosphatase inhibitors (Roche), as well as 250 U/ml benzonase (Sigma). Lysates were rotated for 30 min at 4°C and subsequently denatured for 5 min at 95°C in the presence of loading buffer. Samples were resolved by SDS-PAGE and transferred to nitrocellulose membranes (GE Healthcare) using a Trans-Blot semidry transfer unit (Bio-Rad). Membranes were blocked in 0.1% Tween/phosphate-buffered saline supplemented with 5% skimmed milk (Sigma) and subjected to immunoblotting with the following primary antibodies at the indicated dilutions: phosphorylated STING Ser^366^ (Cell Signaling Technology; 1:1,000), STING (Cell Signaling Technology; 1:1,000), cGAS (Cell Signaling Technology; 1:1,000), IRF-3 (Abcam; 1:1,000), phosphorylated IRF-3 Ser^386^ (Abcam, 1:1,000), α-tubulin (Upstate Biotech; 1:10,000), and D8 (a gift from David Ulaeto [Defence Science and Technology Laboratory, United Kingdom]). Primary antibodies were detected using IRDye-conjugated secondary antibodies in an Odyssey infrared imager (LI-COR Biosciences). Images were analyzed using Odyssey software, and data were obtained after integration of at least three independent experiments.

### STING dimerization.

Analysis of STING dimerization was performed as described previously (29) with minor modifications. THP-1-IFIT-1-GLuc cells were seeded in 24-well plates at a density of 2 × 10^5^ cells per well in the presence of PMA (50 ng/ml). After 48 h, the cells were infected with the indicated viruses in RPMI 1640 supplemented with 2% FCS at the indicated PFU/cell. After 6 h, the cells were transfected with HT-DNA at 4 μg/ml for 4 h or exposed to 15 μg/ml cGAMP for 6 h and lysed as described above, with the exception that lysates were mixed with NuPAGE LDS sample buffer (Bio-Rad) and directly analyzed by SDS-PAGE.

### Statistical analysis.

Statistical significance was determined using an unpaired Student *t* test with Welch's correction where appropriate using GraphPad Prism statistical software.
